# Plant G-Proteins Come of Age: Breaking the Bond with Animal Models

**DOI:** 10.3389/fchem.2016.00024

**Published:** 2016-05-24

**Authors:** Yuri Trusov, José R. Botella

**Affiliations:** School of Agriculture and Food Sciences, University of QueenslandBrisbane, QLD, Australia

**Keywords:** heterotrimeric G-proteins, plant signaling, receptor-like kinases, plant defense, control of plant development, extra-large G-proteins

## Abstract

G-proteins are universal signal transducers mediating many cellular responses. Plant G-protein signaling has been modeled on the well-established animal paradigm but accumulated experimental evidence indicates that G-protein-dependent signaling in plants has taken a very different evolutionary path. Here we review the differences between plant and animal G-proteins reported over past two decades. Most importantly, while in animal systems the G-protein signaling cycle is activated by seven transmembrane-spanning G-protein coupled receptors, the existence of these type of receptors in plants is highly controversial. Instead plant G-proteins have been proven to be functionally associated with atypical receptors such as the Arabidopsis RGS1 and a number of receptor-like kinases. We propose that, instead of the GTP/GDP cycle used in animals, plant G-proteins are activated/de-activated by phosphorylation/de-phosphorylation. We discuss the need of a fresh new look at these signaling molecules and provide a hypothetical model that departs from the accepted animal paradigm.

## Introduction

Heterotrimeric G-proteins (G-proteins) are universal signal transducing proteins that, in animals, mediate signaling from G-protein coupled receptors (GPCRs). Most G-protein research has been concentrated in humans where they play crucial roles in a multitude of cellular and developmental pathways (Simon et al., 1991). The scientific interest on G-proteins can be easily stated by numbers: since Alfred Gilman and Martin Rodbell received the Nobel prize in 1994 for their discovery (1994)^1^, there have been in excess of 22,000 publications in peer-reviewed journals dealing with G-proteins or their associated GPCRs, of which, only an infinitesimal part are devoted to plant G-proteins (< 350). In view of the vast amount of knowledge accumulated in animal systems it is not surprising that the “animal model” became “canonical.” Therefore, from the very beginning, plant G-proteins have been modeled on their animal counterparts and, most importantly, studied as an extension of the animal paradigm.

The G-protein functional complex is conserved across plants and animals and consists of three subunits (Gα, Gβ, and Gγ). In animal systems, activation of the 7-transmembrane-spanning GPCRs, promote the exchange of GDP for GTP in Gα, causing a conformational change that leads to activation of the heterotrimer accompanied or not by dissociation of the Gα subunit from the Gβγ dimer. Gα and the Gβγ dimer then transmit the signal to their specific effector molecules until the intrinsic GTPase activity of the Gα subunit hydrolyses the GTP molecule, returning Gα to its inactive state and sequestering Gβγ back to the inactive heterotrimer. This return to basal state is accelerated by multiple Regulators of G Signaling proteins (RGS) (Siderovski and Willard, 2005).

Although, nobody dared to openly admit it, and thus confront the animal research “big brothers,” given the extraordinary amount of evidence supporting the established animal system, there were numerous signs from the very beginning that plants and animals have followed different “G-protein paths.” Here we discuss the long and winding road that plant G-proteins have taken on their way through puberty and finally independence from their “animal relatives.”

## Unlike animals, there are very few canonical G-protein subunits in plants

In animal systems, G-proteins mediate the signaling of over 800 agonist-activated GPCRs (Pierce et al., 2002). Multiple family members exist for each of the three subunits (23 Gα, 5 Gβ, 12 Gγ in humans) and different combinatorial possibilities provide the required specificity for multiple G-protein based signaling pathways (Wettschureck and Offermanns, 2005). In contrast, plants have a limited set of subunits, with a single Gα (GPA1) and Gβ (AGB1) subunits and two canonical Gγ subunits (AGG1 & AGG2) in Arabidopsis (Ma et al., 1990; Weiss et al., 1994; Mason and Botella, 2000, 2001). In rice, the repertoire of canonical subunits is even smaller with a single isoform for each subunit (Kato et al., 2004; Trusov et al., 2012).

## Too few subunits, too many pathways

The availability of mutants for all canonical G-protein subunits provided a powerful tool for functional and genetic studies. The numerous characterization studies published link G-proteins to a surprisingly wide variety of plant processes including defense (Llorente et al., 2005; Trusov et al., 2006, 2009, 2010), morphological development (Ullah et al., 2001; Goubaeva et al., 2003), cell proliferation (Crespo et al., 1994; Ullah et al., 2001; Chen et al., 2006), ion-channel regulation (Armstrong and Blatt, 1995; Wang et al., 2001), stomatal control (Assmann, 1996; Cheung et al., 2008), light perception (Warpeha et al., 1991, 2007; Okamoto et al., 2001) early seedling development (Lapik and Kaufman, 2003) and phytohormone responses including abscisic acid (ABA), gibberellins (GA), brassinosteroids (BR), ethylene, jasmonic acid (JA), and auxins (Ullah et al., 2003; Chen J. G. et al., 2004; Chen Y. L. et al., 2004; Mishra et al., 2006; Trusov et al., 2006; Okamoto et al., 2009). The involvement of G-proteins in so many pathways was puzzling given that the small number of possible heterotrimer subunit combinations could not provide the required specificity, an essential requirement for signaling pathways. Different stimuli require different signaling pathways to elicit specific developmental and cellular responses. In humans, the large number of available G-protein subunits can provide enough combinatorial possibilities to provide specificity for a large number of stimuli but in plants this was not the case as the initial set of canonical subunits in Arabidopsis (1 Gα, 1 Gβ, and 2 Gγs) was very limited (before the additional non-canonical subunits were added to the repertoire).

Our group proved that the different Gγ subunits confer some level of functional selectivity to the Gβγ dimer signaling in Arabidopsis, but the three functional subunits initially available, namely Gα, Gβγ_1_, and Gβγ_2_, could not provide specific signaling for all the G-protein dependent pathways (Chakravorty and Botella, 2007; Trusov et al., 2007). Most importantly, the signaling specificity provided by the Gγ subunits is partially provided through transcriptional regulation instead of residing on the structural properties of the subunits themselves. In some cellular processes AGG1 can complement AGG2-deficient mutants if it is expressed in the correct tissues and vice versa (Thung et al., 2013). Suspicions were accentuated when we proved that a double mutant *agg1 agg2*, lacking both canonical Gγ subunits did not phenocopy the *agb1* mutation as it was expected from the animal-based canonical model (Trusov et al., 2008).

## Plants have G-protein subunits with unique structures not seen in animals

The “Gγ1 + Gγ2 ≠ Gβ” paradox (Trusov et al., 2008) could only be explained by the existence of additional, yet undiscovered, subunits in the Arabidopsis genome or, alternatively, the possibility that AGB1 could work alone without the need to form a dimer. Given the very high affinity that AGB1 has for each of the AGG subunits, it was difficult to conceive the existence of unbound subunits in the cell but extensive searches of the fully sequenced Arabidopsis genome failed to identify any additional canonical G-protein subunits (Trusov et al., 2008). There was nevertheless an unlikely candidate hiding deep in the genome; a protein containing an N-terminal domain with homology to Gγ subunits followed by a putative transmembrane domain and a large cysteine-rich C-terminal region. Against all expectations, this new protein proved to be a *bona fide* Gγ subunit (AGG3), located in the plasma membrane and showing a strong interaction with AGB1(Chakravorty et al., 2011). AGG3-deficient mutants accounted for all but one of the “orphan” phenotypes unexplained by the two canonical Gγ subunits and the triple *agg1 agg2 agg3* mutant recapitulated all the phenotypes known for *agb1* mutants so far (Thung et al., 2012). Interestingly, AGG3 homologs had been known in rice for some time although, given the radical differences with their canonical animal counterparts, they had not been effectively identified as G-protein subunits (Chakravorty et al., 2011). Not one but two AGG3 homologs had been cloned in rice after physical mapping of two important yield-related QTLs, GS3, a major QTL for grain length and weight and DEP1, a QTL for grain number per panicle and panicle density (Fan et al., 2006; Huang et al., 2009). The discovery of AGG3 not only helped to explain discrepancies in Arabidopsis but also provided a mechanistic model to explain the possible mode of action of the GS3 and DEP1 QTLs (Botella, 2012).

The Arabidopsis AGG3 and its rice homologs radically depart from all accepted canonical features of animal Gγ subunits. While animal Gγs are characterized for being small proteins (~100 amino acids), AGG3 is more than double the size and DEP1 is more than four times larger. In addition, the abundance of cysteine residues present in the C-terminal region of AGG3, GS3, and DEP1 has never before been described in any animal Gγ subunit. Finally, the possibility of a G-protein subunit spanning the plasma membrane was unheard of in animal systems and, even though the presence of a transmembrane domain was only suggested in the initial characterization work, it has now been firmly established for AGG3 (Wolfenstetter et al., 2014). All the AGG3 features places it in a completely different category and opens the door to new and exciting possible signaling mechanisms. AGG3 expands the plasma membrane with the γ domain in the cytosol, able to associate with AGB1 and thus transmit signaling through Gβγ3 dimers while its extracellular cysteine-rich domain is free to interact with extracellular domains from receptors or perhaps bind agonists by themselves, without the intervention of receptors. Interestingly, the rice AGG3 homolog (DEP1) has been located to the plasma membrane and the nucleus suggesting that in this case the protein might not span the membrane adding yet another twist to the story (Huang et al., 2009; Sun et al., 2014).

## Some plant G-protein γ subunits are missing essential animal components

The structural differences between plant and animal G-protein subunits are not limited to the addition of extra domains. In a recent study, Trusov et al. (2012) showed that many plant Gγ subunits are missing an essential component of animal Gγs, the C-terminal CaaX motif. The CaaX motif, where “C” is a cysteine, “a” is preferably an aliphatic amino acid and “X” can be any residue, is essential for prenylation. Animal Gγ subunits undergo post-translational modification by prenylation and proteolytic cleavage of the last three amino acids. The addition of a farnesyl or geranylgeranyl group to the cysteine residue of the CaaX motif allows Gγ to anchor the Gβ subunit (and thus the Gβγ dimer) to the plasma membrane, an absolute functional requirement in animals (Gautam et al., 1998; Takida and Wedegaertner, 2003). Trusov et al. (2012) classified plant Gγ subunits into three types; type A conforms with all the known requirements for animal Gγ subunits, type B lacks the CaaX motif and type C has the additional C-terminal cysteine-rich domain first observed in AGG3 (Trusov et al., 2012). The N-terminal CaaX, this motif is essential for localization and function of plant type A subunits (Chakravorty and Botella, 2007; Zeng et al., 2007). Interestingly, Arabidopsis and the rest of the Brassicaceae family lack type B subunits (AGG1 and AGG2 are both type A subunits), resulting in an almost complete lack of information about these Gγ subunits. Only recently, a type B Gγ subunit has been studied in tomato and silencing of the gene in transgenic lines resulted in hypersensitivity to auxins concomitant with strong hyposensitivity to ABA during germination (Subramaniam et al., 2016).

## Plant vs. animal canonical Gα subunits: Same structure - different kinetics

The Arabidopsis GPA1 subunit shows strong sequence homology with animal Gα subunits; with the closest homologs being the rat inhibitory guanine nucleotide-binding regulatory factors α subunits G_*i*1−3_ and the bovine rod transducing (36% amino acid identity and 73% similarity; Ma et al., 1990). GPA1's crystal structure is almost identical to the human inhibitory Gα protein but amazingly their kinetic properties are completely different (Urano et al., 2013).

In open contrast with animal Gαs, GPA1 spontaneously releases GDP and binds GTP without the need for a GPCR to catalyze the exchange (Johnston et al., 2007a; Jones et al., 2011a). In addition GPA1 has a very low GTPase activity with a catalytic constant 30–100 times smaller than the human Gαs (Graziano et al., 1989; Johnston et al., 2007a). The high rate of non-catalyzed exchange of GDP for GTP combined with the low GTPase activity has led to the suggestion that GPA is constitutively active in the cell by default, exactly the opposite than animal Gαs (Johnston et al., 2007a; Jones et al., 2011a). The kinetic properties of the Arabidopsis GPA1 are not an isolated case and seem to be the norm for other plant Gαs (Urano et al., 2012).

## Extra-large G-proteins (XLGs): The newly discovered G-protein α subunits

The Arabidopsis genome contains three genes encoding proteins with limited homology to Gα subunits but more than twice the size of GPA1 and where thus named extra-large G-proteins (XLGs) upon their discovery (Lee and Assmann, 1999). XLGs contain two distinct domains, a N-terminal cysteine-rich region followed by a C-terminal Gα-like domain (Lee and Assmann, 1999). XLGs have been known for a long time but they have never been considered components of the G-protein heterotrimer for a number of important reasons. In first place, the kinetic and biochemical characteristics of XLGs are quite different from those of canonical Gαs. Even though XLGs can bind and hydrolyze GTP, they use Ca^2+^ as cofactor while Gαs preferentially use Mg^2+^ (Heo et al., 2012). In addition, XLG's affinity for GTP is relatively low and the hydrolysis rate slow even when compared to canonical plant Gαs (Heo et al., 2012). The differences in kinetic and catalytic properties made it difficult to accept XLGs as *bona fide* members of the G-protein heterotrimer but the critical reason for the scientific community to discard XLGs as possible heterotrimer components was the fact that XLGs were initially localized exclusively to the nucleus (Ding et al., 2008), precluding any possible involvement in G-protein signaling which takes place at the plasma membrane. In fact XLG2 was reported to physically interact with the nuclear protein Related To Vernalization 1 (RTV1), enhancing the DNA binding activity of RTV1 to floral integrator gene promoters and resulting in flowering initiation (Heo et al., 2012).

On the other hand, *xlg* mutants share some similar phenotypes with *agb1* mutants suggesting the existence of functional similarities between XLGs and Gβ. For instance, *xlg3* and *agb1* mutants are slightly impaired in root gravitropic responses (Pandey et al., 2008), triple *xlg1 xlg2 xlg3* mutants have longer roots than WT, as observed in *agb1* mutants (Ding et al., 2008) and *xlg2* mutants displayed increased susceptibility to *Pseudomonas syringae*, suggesting a role in plant defense as previously established for AGB1 (Zhu et al., 2009).

Our recent report has now firmly established XLGs as genuine members of the G-protein heterotrimer (Maruta et al., 2015). We have provided genetic proof that XLGs and the Gβγ dimer are involved in the same signaling pathway mediating plant defense. We also provided incontrovertible evidence that XLGs, aside from being located in the nucleus, as previously reported, are also found at the plasma membrane opening the door for a functional role for XLGs within the G-protein signaling heterotrimer. Indeed, we established that there is physical interaction between XLGs and the Gβγ dimer and the interaction is confined to the plasma membrane and not detected in the nucleus. Our findings were later confirmed (Chakravorty et al., 2015) extending the study to include other physiological traits.

Even though the establishment of XLGs as *bona fide* members of the G-protein heterotrimer answers many important questions, it also creates new ones such as whether the Gα_GPA1_βγ and Gα_XLG_βγ heterotrimers share the same activation mechanism. The strong differences observed in the GTP-associated kinetics between GPA1 and XLGs in Arabidopsis makes it unlikely that they share the same activation/deactivation mechanism, unless the GTP-GDP cycle is not the determining factor controlling G-protein activity in plants.

## Plant and animal systems have different G-protein-associated receptors

While in animals G-proteins are associated almost exclusively with GPCRs, in plants they have diversified their signaling capabilities to mediate signals from other receptor families. In fact, the existence of prototypical, animal model, GPCRs in plants is highly controversial and has been hotly contested (Urano and Jones, 2013; Taddese et al., 2014). The first candidate GPCR reported in plants was the Arabidopsis GCR1 with claims that it was a cytokinin receptor (Plakidou-Dymock et al., 1998), an assertion that was promptly disputed (Humphrey and Botella, 2001) and eventually led to a retraction by the authors (Kanyuka et al., 2001). Another GPCR, GCR2 was identified as an ABA receptor (Liu et al., 2007) but was also strongly contested (Gao et al., 2007; Johnston et al., 2007b). Independently of their possible roles as hormonal receptors, plant GPCR candidates have been mostly identified through bio-informatics analysis using structural characteristics such as having seven-transmembrane-spanning (7TM) domains, instead of more important functional attributes such as having guanine nucleotide exchange factor (GEF) activity (Chung et al., 2011). In order for a receptor to be a GPCR it needs to transmit the signal through G-proteins and in the case of GCR1 it was reported that it physically interacts with GPA1 (Pandey and Assmann, 2004), a claim that other authors could not reproduce (Urano and Jones, 2013). In summary, although yet another bioinformatics analysis has recently supported GCR1's identity as a GPCR (Taddese et al., 2014), things are not looking well for the prototypical animal model 7-transmembrane spanning GPCRs in plants.

One receptor proven to be associated with G-proteins in Arabidopsis is RGS1, a protein containing a predicted 7TM domain and a regulator of G-protein signaling (RGS) domain with GTPase accelerating activity at the C-terminus. RGS1 has been proposed to keep the plant G-protein complex in its inactive state and *rgs1* mutants display increased Gα activity (Chen et al., 2003). Upon binding of an agonist, RGS undergoes phosphorylation and subsequent endocytosis, releasing the G-protein complex, which spontaneously activates (i.e., loads with GTP) starting the signaling cycle (Jones et al., 2011b). Although, RGS's 7TM topology is evocative of animal GPCRs, its RGS functional domain makes it unique since animal RGS proteins are not structurally linked to receptors. It is important to keep in mind that although RGS1 has been linked to some G-protein mediated processes such as D-glucose signaling, it does not seem to be involved in most of the G-protein mediated signaling.

Aside from the receptors discussed above, plant G-proteins have been associated with a number of receptors lacking 7TM domains. The maize canonical Gα subunit mediate signaling from FEA2, a CLAVATA LRR receptor (a single pass transmembrane protein), and similar results were reported in Arabidopsis for the GPA1 subunit (Bommert et al., 2013; Ishida et al., 2014). G-proteins play an important role in the plant innate immune response and while the canonical Gα subunit is not involved, the Gβγ dimer and two different XLGs have been proven to mediate signaling in the pathogen-associated molecular patterns-triggered immunity (PTI) (Trusov et al., 2006, 2009, 2010; Chakravorty et al., 2012; Trusov and Botella, 2012; Maruta et al., 2015). Many of the receptors associated with PTI are receptor-like kinases (RLKs) and it was recently proven that the Gβγ dimer mediate signaling by at least three defense related RLKs (Liu et al., 2013). Meanwhile, XLGs have also been functionally linked to several RLKs (Maruta et al., 2015). Aside from the genetic interaction, we have now evidence of physical interaction between G-protein subunits and several RLKs suggesting that G-protein signaling occurs immediately after recognition of the signals by the receptors (Aranda-Sicilia et al., 2015). Recent evidence has linked G-proteins to the mitogen activated protein kinase signaling cascade through the scaffold protein RACK1 in a novel plant immune pathway activated by pathogen secreted proteinases (Cheng et al., 2015). In fact RACK1 had previously been identified as a g-protein interactor (Klopffleisch et al., 2011). It is not yet known which receptors initiate this newly discovered signaling but it would not be surprising if it is a RLK as is the case with other PAMP signaling. The association of G-proteins with RLKs is not limited to plant defense as they mediate signaling from RLKs during nodulation in soybean and physically interact with the LysM-type receptor kinases GmNFR1α and GmNFR1β (single pass transmembrane RLKs; Indrasumunar et al., 2011; Choudhury and Pandey, 2013).

## A new hypothesis: In plants, activation/deactivation of G-proteins is controlled by phosphorylation instead of GTP/GDP exchange (in most cases)

**Figure 1:**
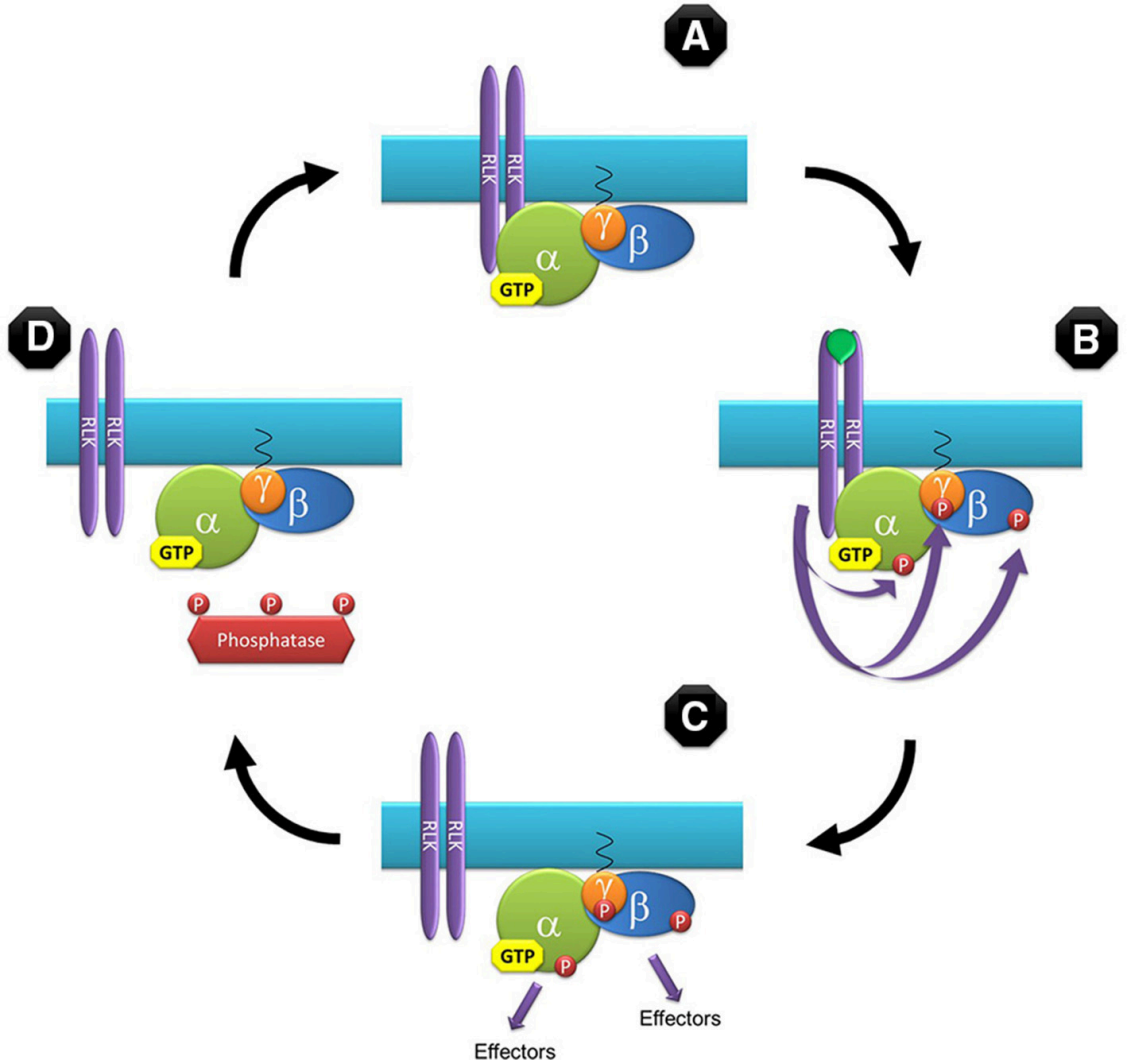
**Model for the G-protein cycle in plants**. This model assumes that Gα is bound to GTP by default based on the reported kinetic properties for GPA1. The GTP-bound heterotrimer has the proper conformation to allow interaction with downstream effectors but is not functionally active **(A)**. Upon binding of an agonist, RLKs (or other associated kinases) phosphorylate the G-protein subunits, activating them and initiating the signaling cycle **(B)**. Signaling proceeds by the two functional subunits (Gα and the Gβγ dimer) **(C)** until the phosphate groups are removed by phosphatases **(D)** rendering the heterotrimer inactive and associating again with a RLK to complete the cycle **(A)**. In this model Gα can be either the canonical (GPA1-like) or non-canonical (XLG-like) subunit.

Our model assumes that Gα subunits are bound to GTP by default (Johnston et al., 2007a; Jones et al., 2011a; Urano et al., 2012). In the classic animal model GTP-bound G-proteins are active by definition but, although not inconceivable, a constitutively active G-protein is not the ideal candidate for a signaling molecule. In this model, binding of GTP to the Gα subunit results in a conformation change in the heterotrimer in such a way that the interacting molecular surfaces of the two functional modules (Gα and Gβγ) are exposed to the downstream effectors (Figure 1A). But in contrast to the animal model we propose that GTP-bound plant G-proteins are not intrinsically active and functional activation is achieved by phosphorylation mediated by protein kinases (Figures 1B,C). Likely candidates to exert this phosphorylation are a number of RLKs that have been proven to physically or genetically interact with G-protein subunits (Choudhury and Pandey, 2013; Liu et al., 2013; Aranda-Sicilia et al., 2015; Maruta et al., 2015), or in the case of non-RLK receptors, their associated kinases (Bommert et al., 2013; Ishida et al., 2014). Termination of signaling in our model is not controlled by hydrolysis of GTP to GDP but by de-phosphorylation mediated by protein phosphatases (Figure 1D). Although, not as prolific as kinases, phosphatases are quite abundant in plants; with 112 phosphatase catalytic subunit sequences identified in the Arabidopsis proteome (Kerk et al., 2002). Originally protein phosphatases were thought to lack specificity and simply balance phosphorylation in a housekeeping mode but recent studies have revealed that many phosphatases are quite specific (Uhrig et al., 2013). Noteworthy, direct interaction between the Arabidopsis Gβ subunit and 2C-type protein phosphatase, PP2C52, has been reported (Tsugama et al., 2012).

**Figure 2:**
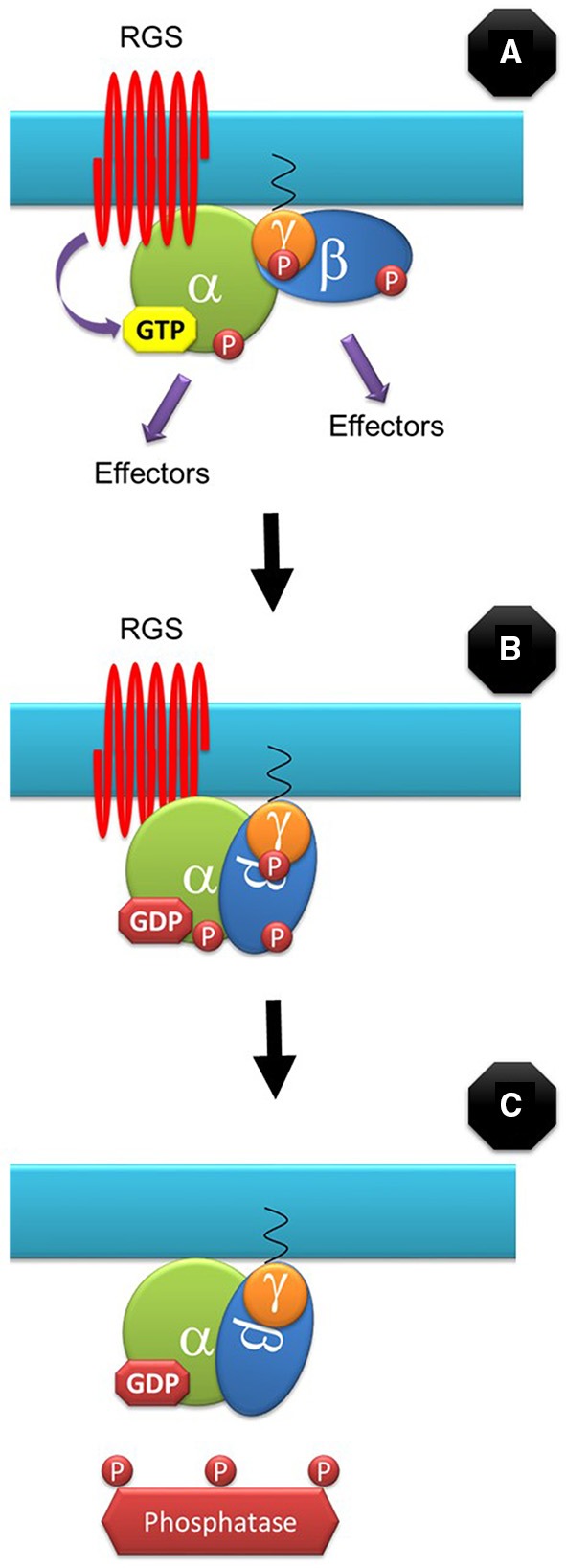
**RGS-dependent signaling control**. For some signaling events, the G-protein cycle can be short-circuited by RGS by stimulating the GTPase activity of the canonical Gα subunit **(A)**. GDP-bound G protein conformation does not allow interaction with downstream effectors and it is therefore inactive, independently of its phosphorylation state **(B)**. Eventually, phosphatases will de-phosphorylate the subunits before they can bind GTP again **(C)**.

Where does RGS1 fit in this model? In our model, a GDP-bound heterotrimer is intrinsically inactive as its conformation does not expose the required molecular surfaces to the downstream effectors and therefore it cannot propagate the signal (independently of its phosphorylation state). We propose that for some processes such as D-glucose signaling, RGS1 can short-circuit the signaling cycle by promoting GTP hydrolysis by the canonical Gα subunits (Figure 2). RGS is not involved in many G-protein-dependent signaling processes such as defense and therefore cannot be the only regulator of the G-protein cycle.

XLGs have not been proposed to be GTP-bound by default as is the case for GPA1 but the model can also be applied to XLG-containing heterotrimers. The GTP binding and hydrolytic activities of Arabidopsis XLGs are much lower than those measured for the canonical GPA1 and it could even be questioned whether they bind GTP *in vivo*, opening the door for a nucleotide-independent signaling cycle for XLGs exclusively controlled by phosphorylation.

Although, our model radically depart from the animal based paradigm, the published literature clearly establishes that plant G-proteins have taken a very different path from their animal counterparts with new structural domains, new subunits and different kinetics, therefore it is not that surprising that they have also evolved a different signaling cycle. G-protein subunits are phosphorylated *in vivo* (Benschop et al., 2007; Heazlewood et al., 2008; Sugiyama et al., 2008; Chen et al., 2010; Nakagami et al., 2010; Aranda-Sicilia et al., 2015) and on-going experiments in our laboratory seem to indicate that substitution of several of the phosphorylated residues with non-phosphorylatable alanines render the subunits inactive and thus unable to restore a wild type phenotype in their respective Arabidopsis mutants.

## “My son, it is time for you to leave home”

In view of the arguments discussed above, we think that it is high time for plant scientists to severe the umbilical cord linking plant and animal G-protein research. We need to put aside the preconceived animal paradigms and study plant G-proteins with a completely open mind. It is true that they have structural homologs in animals but, aside from the fact that they both signal just beneath the cell surface, they are just distant relatives living in a faraway country, with different customs, and more importantly a different language.

Exciting times are ahead of us. New associated receptors, activation mechanisms and signaling pathways need to be established for plant G-proteins. And we believe that the bag of surprises is far from empty……

## Author contributions

All authors listed, have made substantial, direct and intellectual contribution to the work, and approved it for publication.

### Conflict of interest statement

The authors declare that the research was conducted in the absence of any commercial or financial relationships that could be construed as a potential conflict of interest.

## Footnotes

^1^The Nobel Prize in Physiology or Medicine (1994). Available online at: http://www.nobelprize.org/nobel_prizes/medicine/laureates/1994/
